# A mathematical model of dietary lipid absorption and postprandial chylomicron dynamics

**DOI:** 10.1016/j.metop.2026.100522

**Published:** 2026-10-30

**Authors:** Christian Simonsson, Oscar Silfvergren, Henrik Podéus, Kajsa Tunedal, William Lövfors, Karin G. Stenkula, Elin Nyman, Gunnar Cedersund

**Affiliations:** a Department of Biomedical Engineering, Linköping University, Linköping, Sweden; b Department of Experimental Medical Science, Lund University, Lund, Sweden; c SUND Sound Medical Decisions, Linköping, Sweden; d Department of Health, Medicine and Caring Sciences, Linköping University, Linköping, Sweden

## Abstract

Obesity and related conditions such as dyslipidaemia impose an increasing burden on healthcare systems worldwide. These conditions are associated with altered postprandial chylomicron (CM) metabolism, which is governed by complex intestinal processes and substantial inter-individual variation. In particular, the second meal effect (SME) involves the release of previously stored fat from enterocytes during subsequent meals. Mathematical models have previously been used to investigate CM and lipid metabolism. However, these models primarily describe TAG dynamics following a single meal or are too complex for practical personalisation across datasets. Herein, we address these limitations by presenting a small-scale mathematical model of CM dynamics that incorporates the SME. The model describes data from six clinical studies of both single and repeated meal interventions. Model performance was further evaluated using independent datasets, with BMI used to parameterise selected model relationships. Finally, to demonstrate model applicability, we simulated full-day responses consisting of three sequential meals in individuals with varying BMI values. The model generated two key predictions: approximately 2-fold higher postprandial CM exposure at BMI 30 than BMI 20 and a diminishing SME with 5–8 h between meals, both broadly consistent with the literature. This work provides a compact model for investigating the SME and postprandial CM responses across different meal interventions.

## 1 Introduction

Obesity and related metabolic conditions, such as insulin resistance, atherosclerosis, and type 2 diabetes (T2D), are placing an increasing burden on healthcare systems. In T2D research, mathematical models of insulin and glucose regulation have substantially advanced our understanding of disease progression and helped facilitate the development of treatment strategies [1], [2]. In contrast, fewer mathematical models have focused on postprandial lipid metabolism. Dyslipidaemia, characterised by elevated circulating lipid levels, is an important feature of metabolic disease progression and may in part be driven by the postprandial appearance of chylomicrons (CM) [3]. As CM dynamics are complex, incompletely understood, and subject to substantial inter-individual variation, the development of robust mathematical models of CM appearance and postprandial metabolism may be important for advancing our understanding and treatment of metabolic diseases. Such models should also remain sufficiently simple to support interpretation and future individualisation.

A mathematical model of postprandial CM dynamics should capture key biological mechanisms. One such mechanism is the intestines' ability to store triglycerides (TAG) for later release, which contributes to a lipid absorption phenomenon known as the ‘second meal effect’ (SME) [4], [5]. The SME describes how subsequent meals can result in higher CM-TAG release compared to the first meal, even when the meals are identical [6]. Several studies have observed the SME in humans [5], [7], [8], [9], [10], [11], demonstrating complex relationships between CM-TAG release, prior meal composition, and the consumption of other macronutrients. For example, Evans et al. showed that a low-fat meal consumed after a high-fat breakfast still triggered CM-TAG release [5]. Moreover, CM-TAG appearance differs between individuals, with reported differences related to sex, insulin sensitivity, and lipid form (emulsion or solid) [12], [13].

Only a handful of mathematical models have been developed to describe CM-TAG release. One example of such a model is the model by Leohr et al. [14], a semi-physiological model that was used to quantify the CM-TAG response to a high-fat meal in both obese and healthy individuals. While this model describes CM-TAG dynamics following a single meal, it does not aim to capture the SME. Other models have described TAG dynamics following a single meal [15], but these do not specifically describe CM-TAG dynamics. Sosa et al. [11] investigated differences in CM appearance and the SME between insulin-sensitive and insulin-insensitive individuals using experimental data from a single study and systems biology analysis. Their analysis included a mathematical model of sequential-meal responses and supported the hypothesis that stored enteral TAG can contribute to rapid CM secretion. However, there is still a need for a simple and robust model capable of capturing the SME and integrating data from different studies, while remaining suitable for individualized parameterization. Herein, we present such a mathematical model (Fig. 1). The model is designed to describe CM plasma appearance from repeated mixed meals across several independent studies, supporting the analysis of the SME and its underlying mechanisms.

**Fig. 1:**
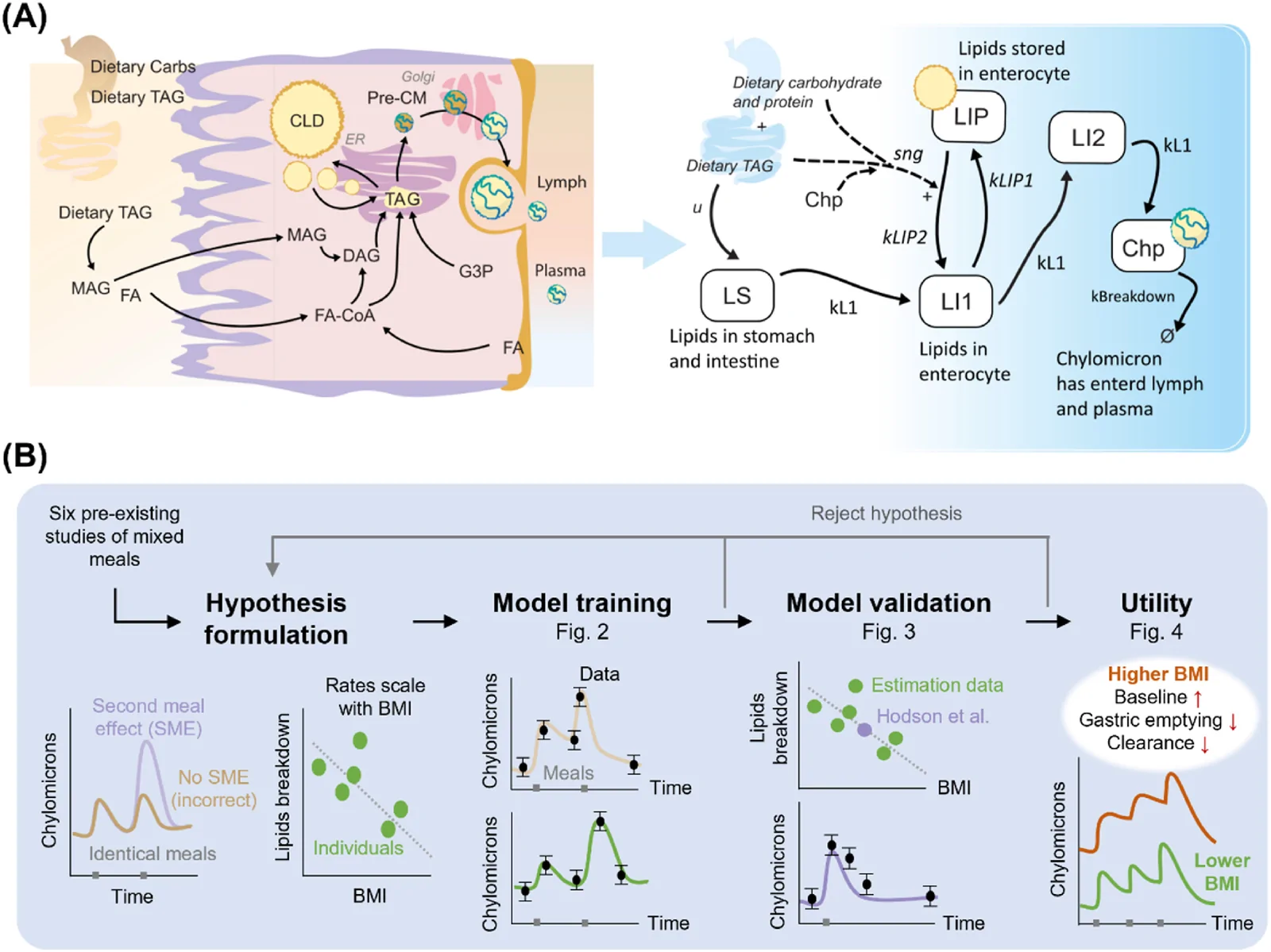
***Study setup and approach. (A)****Physiological processes involved in enterocyte lipid absorption from the intestine (left) translated into a mathematical model (right)*. ***(B)****Methodology and workflow. The model development was conducted in stages: Hypothesis formulation, where a hypothesis was translated into a mathematical model; Model training, in which the model was fitted to data from six heterogeneous studies both individually and jointly; Model validation, where the model's ability to predict independent data was tested; and Utility, where the model was used to predict various repeated meal interventions for individuals with different BMI values*.

## 2 Results

In this study, we have iteratively developed and evaluated a mathematical model for plasma CM-TAG dynamics following both single and multiple meals (Fig. 1A). As a first step, the model was fitted separately to data from six studies (Fig. S1), where two model parameters exhibited substantial interpopulation variability (Fig. S2–S3). As a second step, the mathematical model was trained to all studies jointly while allowing group-specific variability (study and intervention) in the two parameters identified with substantial variability (Fig. 1B, model training). Both parameters were found to be negatively associated with the mean body mass index (BMI) reported for the corresponding cohorts, using linear regression. As a third step, this relation between BMI and model parameters was used to predict CM dynamics of an independent dataset not used in model training (Fig. 1B, model validation). Finally, the established model was used to investigate repeated sequential meals in individuals with varying BMI values (Fig. 1B, utility).

### 2.1 Individual model agreement with CM dynamics for single and multiple meals

The mathematical model of CM postprandial dynamics can describe six different studies individually (Fig. S1). Details regarding each intervention and cohort in the training dataset can be found in the supplementary material (Table S1–S3). The agreement between model simulations and data was evaluated using χ^2^ goodness-of-fit tests at a 5% significance level (see Methods). The model passed the χ^2^-test for all studies individually (Table 1). We also investigated the model's ability to predict chylomicron dynamics in conditions different from training data (Fig. S8). This analysis was performed using Barrows et al. dataset has training data which has a pronounced SME. However, the model predictions fail to pass a χ^2^-test for most studies, potentially due to differences in experimental conditions across studies (Table S1–S3). We therefore performed a parameter analysis to better understand the sources of these discrepancies.

**Table 1:** Model agreement when fitted separately to each dataset, with the corresponding χ^2^-test.

Study	χ^2^-test
Pramfalk et al.*,*[12] males (Fig. S1A)	0.40<*T*_*χ2*_ = 21
Pramfalk et al.*,*[12] females (Fig. S1B)	0.28<*T*_*χ2*_ = 21
Milan et al.*,*[16] young (Fig. S1C)	0.05<*T*_*χ2*_ = 12.6
Milan et al.*,*[16] old (Fig. S1D)	0.83<*T*_*χ2*_ = 12.6
Evans et al., [5] low-fat (Fig. S1E)	8.36<*T*_*χ2*_ = 18.3
Evans et al., [5] water (Fig. S1F)	9.16<*T*_*χ2*_ = 18.3
Heath et al.*,*[17] healthy (Fig. S1G)	12.2<*T*_*χ2*_ = 18.3
Vors et al.*,*[13] normal – lipid spread (Fig. S1H)	5.23 <*T*_*χ2*_ = 17.0
Vors et al.*,*[13] normal – lipid emulsion (Fig. S1I)	6.51<*T*_*χ2*_ = 17.0
Vors et al.*,*[13] obese – lipid spread (Fig. S1J)	0.69<*T*_*χ2*_ = 17.0
Vors et al.*,*[13] obese – lipid emulsion (Fig. S1K)	4.87*<T*_*χ2*_ = 17.0
Barrows et al., [9] healthy (Fig. S1L)	2.39<*T*_*χ2*_ = 31.4

A parameter analysis was performed to evaluate the interstudy differences (Fig. S2–S3, Fig. S9–S10). We found that two parameters displayed substantial interstudy variability: *kBreakdown*, governing the rate of CM clearance; and *kL1*, governing the rate of transport through the intestine and enterocytes. This assessment was performed by comparing the parameter boxplot distributions (Fig. S2) and parameter-to-parameter correlations (Fig. S3). For the boxplot distributions, the confidence intervals (CIs) for both *kL1* and *kBreakdown* appeared broader for interventions involving single meals than for interventions involving multiple meals. For the parameter correlations, the analysis indicated that *kL1* and *kBreakdown* were correlated, and thus both could potentially be represented by a shared relationship. Together, these insights indicate that intervention and study variability could be described by allowing freedom in these two identified parameters.

By allowing group-specific variation in the parameters *kBreakdown* and *kL1,* the model can simultaneously describe all estimation data (Fig. 2), supported by a χ^2^-test with 95% confidence interval (110.8 *<T*_*χ2*_ = 148.8). Additional tests were performed to evaluate whether variation in only one parameter was sufficient to represent interstudy differences (Fig. S4 freedom only in *kBreakdown;* Fig. S5, freedom only in *kL1*). We found that freedom in only one of these parameters could pass the χ^2^-test (*kBreakdown*, 147.3 *< T*_*χ2*_ = 148.8; and *kL1,* 128.0 *< T*_*χ2*_ = 148.8). However, neither case was considered acceptable, as they did not sufficiently capture the SME when the meal responses were assessed qualitatively (Fig. S4–S5). Therefore, to more accurately represent interstudy variability, model development proceeded with allowed freedom in both parameters (Fig. 2).

**Fig. 2:**
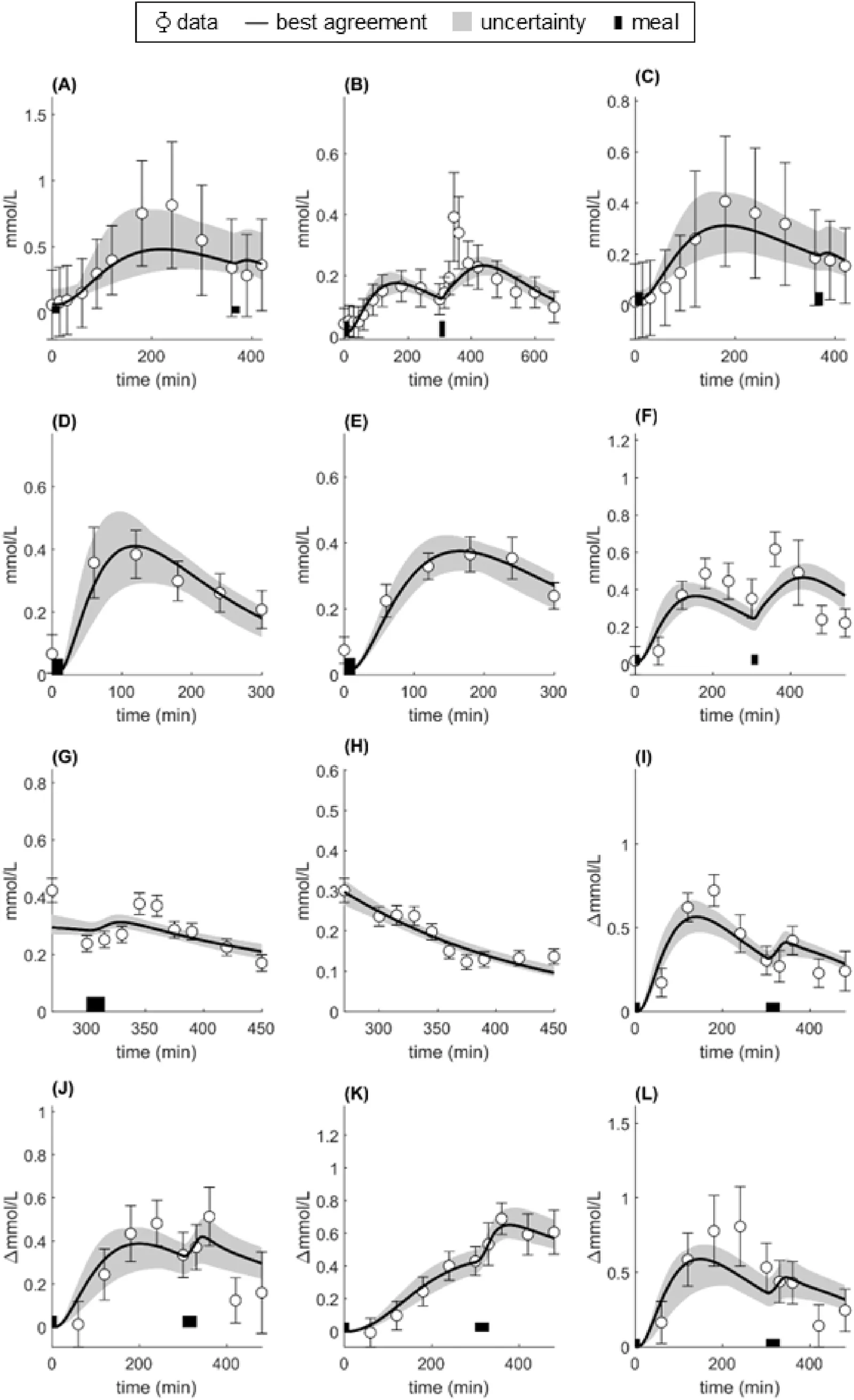
***Model simultaneous agreement to all estimation data*.***For all panels: area indicates model uncertainty, error bars represent data, lines represent model best agreement with data, and thick black bar(s) on x-axis indicate meal consumption*. ***(A)****Pramfalk* et al., [12]*males****(B)****Barrows* et al.*,*[9]*healthy.****(C)****Pramfalk* et al., [12]*females*. ***(D)****Milan* et al., [16]*young,****(E)****Milan* et al., [16]*old*. ***(F)****Heath* et al. [17]*healthy*. ***(G)****Evans* et al. [5]*low-fat*. ***(H)****Evans* et al. [5]*water.****(I)****Vors* et al. [13]*normal spread.****(J)****Vors* et al. [13]*normal emulsion*. ***(K)****Vors* et al. [13]*obese spread.****(L)****Vors* et al. [13]*obese emulsion*.

### 2.2 Model prediction of CM appearance in an independent validation dataset

Values of the two parameters describing study- and population-differences (*kBreakdown* and *kL1*) must be specified to predict CM dynamics in new individuals or meal interventions (Fig. 3). To address this, the relationship between the study-specific parameters and known study covariates, e.g., *BMI,* age, and *Homeostatic Model Assessment for Insulin Resistance* (HOMA-IR) (Table S1), was assessed via a linear-regression analysis (Fig. 3A–B). This was performed using the log-transformed parameter-value (log_e_) corresponding to the best agreement between model and data. We found that the highest correlation was observed between BMI and kBreakdown with a Pearson correlation coefficient (r) of 0.63 (Fig. 3A). A slightly smaller correlation coefficient was observed between BMI and kL1 (r = 0.51) (Fig. 3B). Using these regression equations, we have a tentative representation for study- and population-differences. These relationships were used to assign plausible parameter values when simulating a new cohort.

**Fig. 3:**
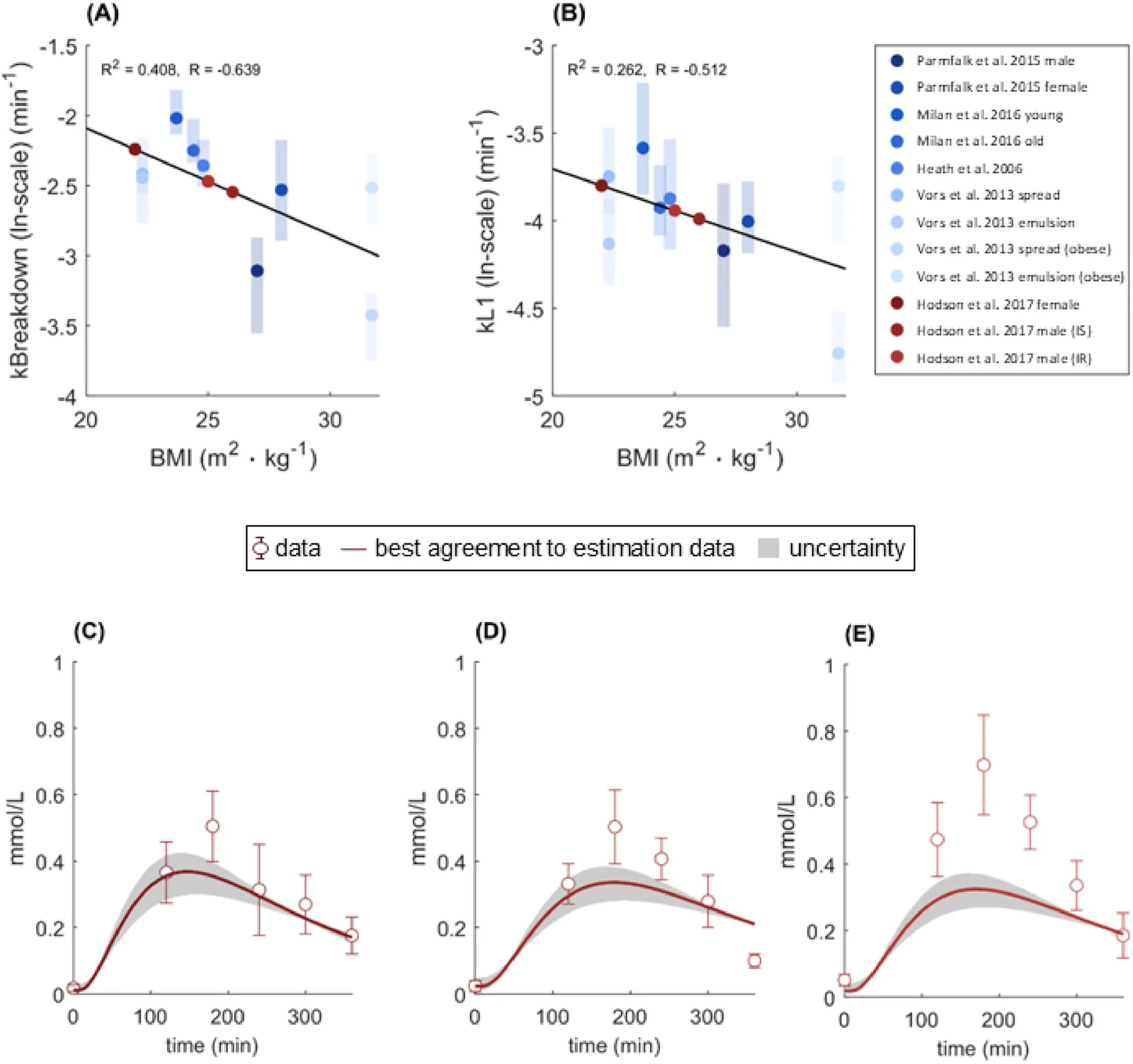
*Model predictions of interventions from an independent study (Hodson* et al.*)*[18]***.****For all panels: area indicates model uncertainty, and error bars represent data.****(A)****The value of kBreakdown for the new cohorts was estimated using the regression model based on BMI. Dots show best found agreement with data. The line shows the fitted linear-regression relationship*. ***(B)****A corresponding regression model was used to estimate the kL1 values. Dots show best found agreement with data. The line shows the fitted linear-regression relationship*. ***(C)****The model prediction of the cohort comprising healthy females from Hodson* et al. [18]***.****Error bars show population data.****(D)****The model prediction of the insulin sensitive men cohort from Hodson* et al. [18]***.****Error bars show population data.****(E)****The model prediction of the insulin-resistant men cohort from Hodson* et al. [18]***.****Error bars show population data.*

We used the BMI-dependent parameterization to make predictions for data from an independent study [18] (Fig. 3C–E). In this study, plasma CM was measured after a single meal in three different cohorts: female (Fig. 3C; mean BMI of 22), insulin sensitive men (Fig. 3D; mean BMI of 26), and insulin resistant men (Fig. 3E; mean BMI of 25). The model simulations agreed with the data from the female cohort (Fig. 3C) and the data from the cohort of insulin sensitive men (Fig. 3D), whereas the model underpredicted the CM concentrations for the insulin resistant men (Fig. 3E). Still, more work is needed to validate the relationship between specific covariates and model parameters.

### 2.3 Model predictions of daily CM variations for different individuals and meal patterns

To highlight the usability of the constructed model, we investigated CM appearance during three sequential meals (breakfast, lunch, and dinner containing 15 g, 30 g, and 30 g of fat respectively) during a day (Fig. 4). The simulations were conducted over several days until a steady state was reached. Using the BMI-dependent parameterization, the model predicted higher CM peaks with increasing BMI levels throughout the day (Fig. 4A). To highlight this behaviour further, we simulated separate CM dynamics for individuals with different BMI values (Fig. 4B) and corresponding mean levels of circulating CM (Fig. 4C), where the model predicts higher daily mean CM for increasing BMI levels (Fig. 4C). More specifically, the model predicts postprandial chylomicron exposure approximately 2-fold higher at a BMI of 30 than at a BMI of 20. This is qualitatively consistent with the literature, where obese individuals (BMI 30 or above) have been observed to have a 2- to 3-fold higher postprandial chylomicron exposure than lean participants [19]. Similar observations have been reported in other works [20], [21].

**Fig. 4:**
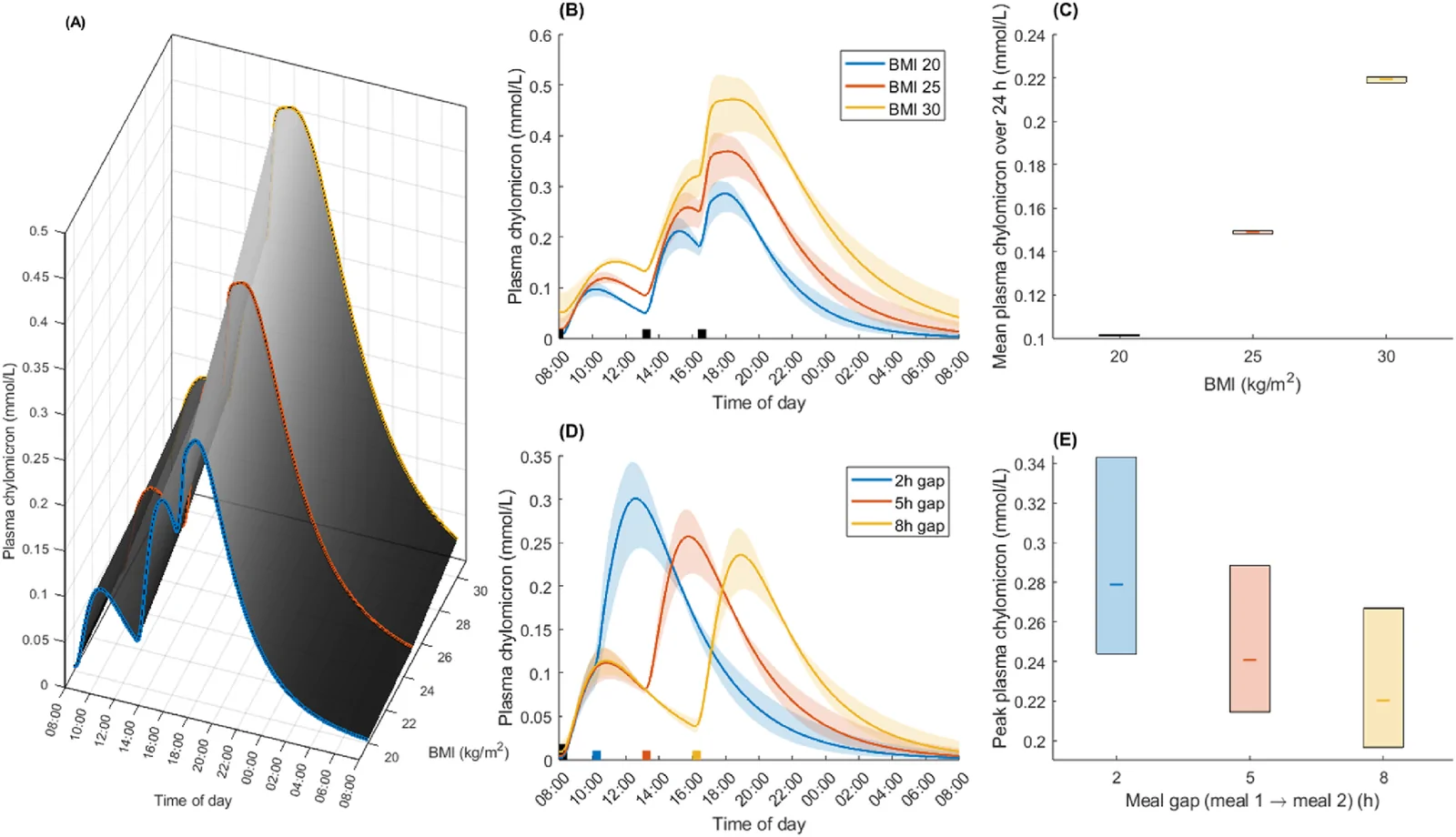
**Model prediction of one day consisting of three meals in individuals with different BMI and time between meals. (A)** Relationship between plasma chylomicron dynamics and BMI over a day of eating three meals. The coloured black area is predicted plasma CM, and coloured lines show three BMI profiles: a BMI of 20 (blue), a BMI of 25 (orange), and a BMI of 30 (yellow). **(B)** Simulations of chylomicron dynamics for three different BMI groups: BMI of 20 (blue), BMI of 25 (orange) and BMI of 30 (yellow). The meals consisted of 15, 30, and 30 g lipids, respectively. **(C)** Predicted mean chylomicron values over the 24-h period shown in Fig. 4B. Lines represent the mean and coloured boxes represent model uncertainty. **(D)** Simulations of chylomicron dynamics for three different groups with different meal timing; 2-h gap from first meal (blue), 5-h gap (orange), and 8-h gap (yellow). The first meal consisted of 15 g and the second of 30 g of lipids **(E)** Predicted peak chylomicron values over the 24-h period shown in Fig. 4D. Lines represent the mean and coloured boxes represent model uncertainty. (For interpretation of the references to colour in this figure legend, the reader is referred to the Web version of this article.)

We also simulated how meal patterns affect the mean level of CM during the day (Fig. 4D–E). The simulations were repeated over multiple days until steady state was reached. The model predicted that increasing the time between two meals (containing 15 g and 30 g fat) reduces the SME, with a reduction between having a 2-h gap and a 5-h gap between meals (Fig. 4D, blue compared to orange). We found that peak of circulating CM decreased with longer time periods between meals (Fig. 4E), with the highest CM peak at 0.34 mmol/L at 2 h between meals, at 0.28 mmol/L for 5 h between meals, 0.26 mmol/L at 8 h between meals. In this meal-timing investigation, an SME is predicted when a meal is consumed 5 h after the first meal (Fig. 4D, orange), which is in agreement with clinical observations [5]. Furthermore, the model predicts a diminishing SME effect between 5 and 8-h (Fig. 4D, orange compared to yellow), which is in qualitative agreement with reports that chylomicron remnants are cleared after approximately 6-8 h after eating [22].

In conclusion of the meal intervention predictions, the model together with the BMI-dependent parameterization shows qualitative agreement with several clinical observations in circulating CM levels and provides a small-scale mathematical model capable of simulating CM postprandial dynamics.

## 3 Discussion

We have constructed a mathematical model describing plasma CM appearance (Fig. 1A) and evaluated its ability to describe data in several steps (Fig. 1B). First, we found that the model could sufficiently describe six different clinical studies individually (Fig. S1; as confirmed using individual χ^2^-test). Second, we found that the most important parameters to describe group variability (study and intervention) were *kL1* (governing enterocyte transport of lipid) and *kBreakdown* (CM clearance from plasma) (Fig. S2–S3). By allowing freedom in these two parameters and restricting variability in all other parameters, the model was able to simultaneously fit all estimation data (Fig. 2), confirmed by a χ^2^-test (110.8 < 148.8 = Tχ^2^). A relationship between the group-specific values of kL1 and kBreakdown and cohort-level mean BMI was found using linear regression (Fig. 3A–B). Third, the established BMI-dependent parameterization was not rejected by independent validation data (Fig. 3C–D). Finally, we investigated how the model could be used to predict postprandial chylomicron dynamics of various meal interventions for individuals with different BMI values (Fig. 4), broadly consistent with the literature. In summary, we have constructed a small-scale mathematical model of CM appearance and the SME, providing a foundation for creating and extending models describing postprandial lipid metabolism.

An important aspect of the model presented is its relatively small number of parameters compared with more comprehensive descriptions of postprandial lipid metabolism [11], as well as its ability to adapt predictions through BMI-dependent parameterization, broadly consistent with the literature (Fig. 4). The low model complexity was intentional, as a compact model facilitates interpretation and integration across studies while providing a practical basis for future individualisation. The ability to describe the SME across multiple independent studies with a limited number of variable parameters suggests that a simplified representation can capture important features of CM dynamics, although additional mechanisms may be required to further explain interindividual and interstudy variability.

The model's ability to predict the SME under various conditions needs to be further evaluated. When the model was trained using only the Barrows et al. [9] dataset, the model could produce a sharp second peak (Fig. S1L), but when trained simultaneously to all studies, the peak is less prominent (Fig. 2B). This is mainly because the SME peak magnitude varies from study to study, for example, between the Vors et al. [13] and Barrows et al. [9] datasets (Fig. 2L compared to Fig. 2B). These discrepancies could be due to several factors not accounted for by the model and, in many cases, not reported in the studies included here, such as differences in how CM was measured, the physical form of the consumed fat (oil, emulsion, or spread), or interpopulation variability not captured by BMI (e.g., insulin sensitivity). While BMI and insulin sensitivity have been reported to correlate significantly [23], we found weak agreement with the insulin resistant group in the Hodson et al. study despite using the BMI-dependent parameterization (Fig. 3E). Future work should evaluate the inclusion of insulin sensitivity as an additional predictor in model predictions to potentially overcome this shortcoming. To further evaluate the predictive ability of the model, we trained the model exclusively on the Barrows et al. dataset and used it to predict all remaining studies (Fig. S8), showing that the model can predict key SME dynamics outside the estimation dataset and revealing remaining between-study variability. With added understanding of how specific study conditions such as e.g., lipid formulation and cohort metabolic health impact model parameters, the models predictive capabilities could be increased. Despite these model limitations and opportunities for future work, the mechanisms incorporated in our small-scale model capture key features of the SME across evaluated studies (Fig. 2, Fig. 3, Fig. 4; as confirmed by individual χ^2^-test and qualitative agreement with literature).

There are several biological mechanisms that were omitted from the presented model. We do not include the production of CMs from plasma-derived *non-esterified fatty acids* (NEFA) and glycerol, nor the conversion of either dietary or plasma-derived carbohydrates into CM-TAG. Furthermore, there are other aspects to expand upon: interactions among gastric emptying, macronutrient composition, and meal volume and the fact that different fatty acids may have different metabolic rates. We recently published an ethanol rate-of-appearance model incorporating a gastric-emptying module [24], and future work could involve integration of such gastric emptying mechanisms to better describe lipid absorption, and interactions with other meal components.

The relationships between covariates such as BMI and HOMA-IR and CM dynamics require further evaluation. Metabolic dysregulation is associated with elevated circulating TAG concentrations, where mechanisms include poor clearance of CM-TAGs, or increased *Very Low Density Lipoprotein* (VLDL)-TAG secretion [25]. The model identified *kBreakdown*, which represents CM clearance, as an important parameter for describing interstudy variability, and we found a tentative cohort-level association between this parameter and BMI (Fig. 3A). The observed cohort-level association between BMI and the model parameters may partly reflect reduced CM clearance associated with impaired insulin sensitivity. Yet, when we tested this correlation, we did not observe a comparable correlation between the breakdown parameter and HOMA-IR (Fig. S6). To further investigate parameter identifiability, a profile-likelihood analysis was conducted (Fig. S9–S10), which indicated adequate identifiability of all model parameters.

There are several additional key aspects to consider in our study. One important consideration is data availability. All data used for model estimation and validation were digitised data from previous works. Therefore, the extracted values may differ slightly from the original numerical data. In many cases substantial covariate information was unavailable (such as BMI, age, and HOMA-IR), which prevented the inclusion of several studies to be included in our regression analysis [5], [9]. Furthermore, another study limitation was the exclusion of total plasma TAG data, which further reduced the number of eligible datasets. By including plasma TAG, the model complexity would also need to increase, to represent additional lipoprotein classes, i.e. VLDL. We have previously developed models for NEFA metabolism [26], lipolysis [27], glucose-insulin regulation [28], [29], [30], and multi-level and multi-timescale models for insulin resistance progression [31], [32]. An integration of all these models into a single framework could be capable of describing mixed meal postprandial metabolism and therefore linking additional mechanisms involved in obesity progression and dietary responses. Herein, we present a step towards such a more complete mathematical model of human meal responses.

In conclusion, we have created a model for plasma CM appearance capable of describing dynamics related to the SME across multiple studies using a BMI-dependent parameterization. The mechanisms governing CM dynamics, including person-specific variability and the effects of the physical form of dietary fat, are still not fully understood, and there appear to be substantial inter-patient and inter-cohort variations in CM dynamics that will need further work to be elucidated. Nevertheless, the presented model provides a practical component that could be integrated into broader mathematical models requiring a description of CM appearance, the SME, and postprandial lipid dynamics.

## 4 Methods

The code to reproduce all reported observations can be found in our GitHub repository (https://github.com/chrsi30/CMDT).

### 4.1 Modelling approach and software

The model was formulated using ordinary differential equations (ODEs). Model formulation, simulation, parameter estimation, and mathematical analyses were performed in MATLAB (2022a; The MathWorks, Natick, MA), using the systems biology toolbox (IQM) [33]. The parameter estimation was performed using the extended scatter search (ESS) optimisation algorithm from the MEIGO toolbox [34]. The quality of the resulting optimal simulations was evaluated using a $\chi^{2}$-test [35]. Finally, the parameter and model uncertainty analysis was done using Markov chain Monte Carlo sampling implemented in the PESTO toolbox [36]. A profile-likelihood analysis was performed to investigate parameter identifiability and uncertainty (Fig. S9–S10). For each parameter, the parameter value was systematically varied over a predefined range while all remaining parameters were re-estimated against the experimental data.

### 4.2 Parameter estimation

Parameter estimation was done by quantifying the model performance, using the model output ŷ to calculate a weighted least squares cost function (Eqs. (Eq. 1), (Eq. 2)).

(Eq. 1) $$\sum_{i}V\left(\theta_{i}\;\right)=\sum_{i}\sum_{j,k}{\left(\frac{y_{,ki}\left(t_{j}\right)-{ŷ}_{i}\left(t_{j,}\theta_{i}\right)}{SEM_{i}\left(t_{j}\right)}\right)}^{2}$$

(Eq. 2) $$SEM=\frac{\sigma_{i}\left(t_{j}\right)}{\sqrt{n_{i}\left(t_{j}\right)}}$$

where θ is the model parameters; $y_{i}\left(t_{j}\right)$ is the measured data from a study *i*, at time point *j and* from one type of measure *k*; ${ŷ}_{i}\left(t_{j,}q\right)$ is the simulation value for a given experiment setup *i* and time point *j*, and SEM is the standard error of the mean, which is the sample standard deviation, $\sigma_{i}\left(t_{j}\right)$ divided by the square root of the number of repeats, $n_{i}\left(t_{j}\right)$ at each time point. The value of the cost function, V(θ), is then minimised by tuning the values of the parameters.

To evaluate the model, a $\chi^{2}$-test was performed for the size of the residuals, with the null hypothesis that the experimental data have been generated by the model. The test was performed under the assumption that the experimental noise is additive and normally distributed [35]. In practice, the cost function value was compared to a $\chi^{2}$-test statistic, $T_{\chi 2\;}^{o}$, with a cumulative density function (Eq. (3)).

(Eq. 3) $$T_{\chi 2\;}^{o}=F_{\chi 2}^{\text{cdf}-\text{inv}}\left(1-\alpha ,v\right)$$

where $F_{\chi 2}^{\text{cdf}-\text{inv}}$ is the inverse density function; and α is the significance level (α = 0.05 was used) and *v* is the degrees of freedom, equal to the number of data points in the training dataset. In practice, the model is rejected if the model cost is larger than the $\chi^{2}$-threshold ($T_{\chi 2\;}^{o}$).

### 4.3 Model description – the chylomicron model

The ODE model was developed through an iterative process of physiologically based, data-driven hypothesis testing. Several candidate model structures were evaluated using the Barrows et al. dataset. The candidates were evaluated based on their ability to accurately describe the experimental data using a χ^2^-test, with special emphasis on the ability to reproduce the sharp second meal peak. Furthermore, the candidate-model complexity was also evaluated based on the Akaike information criterion (AIC) (Table S2). The chosen model (‘M7’) was selected based on its balance between goodness of fit, model complexity, AIC/AICc, and its ability to reproduce the sharp second-meal peak. There are other candidates which performed similarly e.g. M5 and M10, but the M7 model was chosen because it had similar performance as M5 but one less transfer state. The agreement between all candidate model structures and the Barrows et al. dataset, including their ability to reproduce the characteristic second-meal peak, is shown in Fig. S7. The model parameter values and initial state values can also be found in Table S4.

The model has a total of six model states and five model parameters (Fig. 1A, right). The first state represents TAG that has entered the stomach and is moving towards the intestine and has notation LS (Eq. (4)).

(Eq. 4) $$\frac{d}{dt}LS=u_{fat}-kL1\cdot \text{LS}\;LS\left(0\right)=0\;\left(mmol\right)$$

where $u_{fat}$ is the model input, the mass of triglycerides from the meal, expressed as mmol (converted from grams using a TAG molar mass of 845 g/mol), and kL1 (min^−1^) is the rate parameter for the absorption and transfer of lipids into and within the enterocytes. TAG is then transported through the enterocytes and prepared for storage or plasma release. This part is represented by the three model states: L1 (Eq. (5)), *LIP* (Eq. (6)), and L2 (Eq. (8))

(Eq. 5) $$\frac{d}{dt}L1=kL1\cdot \text{LS}-\text{kL}1\cdot L1-kLIP1\cdot L1+\text{LIP}\cdot \left(kLIP2+\text{sng}\right)\;L1\left(0\right)=0\;\left(mmol\right)$$

where kL1 is reused from (Eq. (4)) to describe the transfer rate moving into the next enterocyte state. In Eq. (5), enterocyte lipid storage is introduced, which is the transfer of TAG into lipid droplets, represented by the state LIP. The transfer into and from the LIP state is represented by the parameters kLIP1 (min^−1^) and kLIP2 (min^−1^) respectively. Here we also introduce the phenomenological effect of the second meal, denoted sng , which affects the efflux from the LIP state.

(Eq. 6) $$\frac{d}{dt}LIP=kLIP1\cdot L1-\text{LIP}\cdot \left(kLIP2+\text{sng}\right)\;LIP\left(0\right)=0\;\left(mmol\right)$$

The sng effect is a model state which has the following derivative (Eq. (7)):

(Eq. 7) $$\frac{d}{dt}sng=\left(u_{fat}+u_{carb}+u_{protein}\right)\cdot \text{Chp}-sng\cdot kd\;sng\left(0\right)=0\;\left(\dim\right)$$

where $u_{carb}$ and $u_{protein}$ are the carbohydrate and protein portions of the meal. Here we also allow for the plasma level of chylomicron (Chp) to increase the magnitude of the SME. The last enterocyte state, L2, has the following derivative:

(Eq. 8) $$\frac{d}{dt}L2=kL1\cdot L1-\text{kL}1\cdot L2\;L2\left(0\right)=0\;\left(mmol\right)$$

where kL1 is the rate of TAG-chylomicron excretion into plasma.

The plasma TAG-chylomicron state is denoted Chp and has the following derivative:

(Eq. 9) $$\frac{d}{dt}Chp=kL1\cdot L2-\text{kBreakdown}\cdot Chp\;Chp\left(0\right)=0\;\left(mmol\right)$$

where kBreakdown (min^−1^) is the breakdown and usage of TAG-chylomicrons in the body. To obtain the concentration in plasma the Chp state is divided by the plasma volume, calculated via the Nadler formula, based on sex, height and weight [37].

A complete table of model parameter values, units, and state initial values is provided in the supplementary material (Table S4).

### 4.4 Data curation

Data were gathered and digitised from a total of seven published studies [5], [9], [12], [13], [16], [17], [18]. Studies included in our digitised dataset all had statements declaring that methods were performed in accordance with local ethical approval, relevant guidelines and regulations, and that informed consent was obtained from participants.

The estimation data includes six of these studies (Table S1), and various interventions. The interventions that were investigated in each study are described in the supplementary material (Table S3). Datasets were eligible if CM-TAG concentrations were measured in relation to a meal and sufficient information was provided about both the test meal and any preceding meal. All data was digitised using the WebPlotDigitizer.

## CRediT authorship contribution statement

**Christian Simonsson:** Writing – review & editing, Writing – original draft, Visualization, Validation, Methodology, Investigation, Formal analysis, Data curation, Conceptualization. **Oscar Silfvergren:** Writing – review & editing, Writing – original draft, Validation, Software, Methodology, Investigation, Formal analysis, Conceptualization. **Henrik Podéus:** Writing – review & editing, Methodology, Investigation, Conceptualization. **Kajsa Tunedal:** Writing – review & editing, Methodology, Conceptualization. **William Lövfors:** Writing – review & editing, Supervision. **Karin G. Stenkula:** Writing – review & editing, Supervision, Funding acquisition. **Elin Nyman:** Writing – review & editing, Supervision, Project administration, Investigation, Funding acquisition. **Gunnar Cedersund:** Writing – review & editing, Supervision, Project administration, Investigation, Funding acquisition, Conceptualization.

## Data availability

Source data used for model validation and parameter estimation are presented in the original publications [5], [9], [12], [13], [16], [17], [18] as figures, and numerical datasets were not publicly available. Numerical values were therefore extracted by digitising the values from figures and then used for model fitting and validation. The resulting digitised datasets, together with all model code and parameter values required to reproduce the analyses, are available in our public repository (https://github.com/chrsi30/CMDT).

## Funding information

GC acknowledges support from the Swedish Research Council (2023-03186, 2023-05460), the Horizon Europe project STRATIF-AI (101080875), VINNOVA (VisualSweden), ALF (RÖ-1001928), and the Exploring Inflammation in Health and Disease (X-HiDE) Consortium - a strategic research profile at Örebro University funded by the Knowledge Foundation (20200017). EN acknowledges support from Zenith and the Swedish Fund for Research without Animal Experiments.

## Declaration of competing interest

Gunnar Cedersund is the owner of SUND sound medical decisions AB (unrelated to the work presented herein). Oscar Silfvergren and Kajsa Tunedal are employees of SUND sound medical decisions AB.
