# Supplementary material for: A mathematical model of dietary lipid absorption and postprandial chylomicron dynamics
Source: Metabol Open. 2026 Oct 30;32:100522. doi: 10.1016/j.metop.2026.100522 (PMC13760088; doi:10.1016/j.metop.2026.100522)
Supplement: Supplementary file 1 [file mmc1.docx]

Supplementary material

**Figure S1. *Model agreement to estimation data fitted to each study individually.*** *For all panels: area indicates model uncertainty, error bars represent data, lines represent model best agreement with data, and thick black bar on x-axis indicate meal consumption.* ***(A)*** *Model agreement for the Pramfalk et al.,* (12) *male dataset.* ***(B)*** *Pramfalk et al.,* (12) *female dataset* ***(C)*** *Milan et al.,* (16) *young* ***(D)*** *Milan et al.,* (16) *old* ***(E)*** *Evans et al.,* (5) *low-fat* ***(f)*** *Evans et al.* (5) *water* ***(G)*** *Heath et al.* (17) *healthy* ***(H)*** *Vors et al.* (13) *normal spread* ***(I)*** *Vors et al.* (13) *normal emulsion* ***(J)*** *Vors et al.* (13) *obese spread.* ***(K)*** *Vors et al.* (13) *obese emulsion* ***(L)*** *Barrows et al.* (9) *two identical meals.*

**Figure S2. Parameter coefficients when model is trained to all estimation data individually.** *Boxplots showing the parameter distribution for model parameters.* ***(A)*** *kL1.* ***(B)*** *kd.* ***(C)*** *kLIP1.* ***(D)*** *kLIP2.* ***(E)*** *kBreakdown.*

**Figure S3. 2D parameter profiles after sampling.** This figure shows the relationships between all parameters during the individual agreement to data. The different colors represent the different studies (**Table S1**), with the blue coloured dots being parameters estimated to single-meal studies, and red dots for studies with sequential meals.

**Figure S4. *Model simultaneous agreement to all estimation data with allowed variability only in parameter kBreakdown, while kL1 was shared across groups.*** *For all panels: area indicates model uncertainty, error bars represent data, lines represent model best agreement with data, and thick black bar on x-axis indicate meal consumption.* ***(A)*** *Pramfalk et al.,* (12) *males* ***(B)*** *Barrows et al.,* (9) *healthy.* ***(C)*** *Pramfalk et al.,* (12) *females.* ***(D)*** *Milan et al.,* (16) *young,* ***(E)*** *Milan et al.,* (16) *old.* ***(F)*** *Heath et al.,* (17) *healthy.* ***(G)*** *Evans et al., low-fat* (5)*.* ***(H)*** *Evans et al., water* (5)*.* ***(I)*** *Vors et al., normal spread.* ***(J)*** *Vors et al., normal emulsion* (13)*.* ***(K)*** *Vors et al., obese spread* (13)*.* ***(L)*** *Vors et al., obese emulsion* (13)*.*

**Figure S5. *Model simultaneous agreement to all estimation data with allowed variability only in parameter kL1, while kBreakdown was shared across groups.*** *For all panels: area indicates model uncertainty, error bars represent data, lines represent model best agreement with data, and thick black bar on x-axis indicate meal consumption.* ***(A)*** *Pramfalk et al.,* (12) *males* ***(B)*** *Barrows et al.,* (9) *healthy.* ***(C)*** *Pramfalk et al.,* (12) *females.* ***(D)*** *Milan et al.,* (16) *young,* ***(E)*** *Milan et al.,* (16) *old.* ***(F)*** *Heath et al.,* (17) *healthy.* ***(G)*** *Evans et al.,* (5) *low-fat.* ***(H)*** *Evans et al.,* (5) *water.* ***(I)*** *Vors et al.,* (13) *normal spread.* ***(J)*** *Vors et al.,* (13) *normal emulsion.* ***(K)*** *Vors et al.,* (13) *obese spread.* ***(L)*** *Vors et al.,* (13) *obese emulsion.*

**Figure S6. Regression analysis between parameters kBreakdown and *kL1* to possible covariates. (A)** *kBreakdown* vs BMI. **(B)** *kBreakdown* vs age. **(C)** *kBreakdown* vs HOMA-IR. **(D)** *kL1* vs BMI. **(E)** *kL1* vs age. **(F)** *kL1* vs HOMA-IR.

**Table S1.** Table giving a brief description of the anthropometric data and clinical parameter characterising the study cohort used for training and validation data.

| **Study name** | **Cohort** | **Age** | **BMI** | **HOMA-IR** |
| --- | --- | --- | --- | --- |
| **Barrows *et al.* 2005** | Healthy (M) (n=6) | 20 –55 | - | - |
| **Evans *et al.* 1998** | Healthy (M/F) (n=6) | 21-44 | 20.8-27.2 | - |
| **Milan *et al.* 2016** | Healthy (M/F) (n = 15) | 67.3±1.5 | 24.4±1.0 | 1.7±0.3 |
| **Milan *et al.* 2016** | Healthy (M/F) (n = 15) | 22.7±0.4 | 23.7±0.8 | 2.0±0.2 |
| **Vors *et al.* 2013** | Healthy (n=9) | 28.3±1.4 | 22.3±0.5 | 0.85±0.14 |
| **Vors *et al.* 2013** | Obese (n=9) | 30.2±2.2 | 31.7±0.3 | 1.69±0.25 |
| **Heath *et al.* 2006** | Healthy (M) (n=8). | 46.4±1.4 | 24.8±0.5 | - |
| **Pramfalk *et al.* 2015** | Healthy (F) (n=11). | 46±6 | 28±3 | 2.6 ±1.5 |
| **Pramfalk *et al.* 2015** | Healthy (M) (n=11). | 46±7 | 27±2 | 2.4±0.8 |
| **Hodson et al. 2007** | Healthy (F) (n=11). | 32 (23–64) | 22 (22–27) | 0.9 (0.4–1.3) |
| **Hodson et al. 2007** | Insulin sensitive (M) (n=11) | 42 (23–54) | 26 (21–31) | 1.1 (0.9–1.4) |
| **Hodson et al. 2007** | Insulin resistant (M) (n=11) | 42 (22–45) | 25 (22–35) | 1.7 (1.5–3.5) |

**Detailed description of model candidates**

**Table S2**. We evaluated several candidate model structures before choosing ‘M7’ as the final model structure that was selected based on agreement with the Barrows et al. dataset, model complexity, and reproduction of the prominent SME. The cost shown here is when the model was fitted to the Barrows et al. 2005 dataset.

| **Model Name** | **Parameters** | **States** | **Cost** | **AIC** | **AICc** |
| --- | --- | --- | --- | --- | --- |
| **M1** | 3 | 4 | 7.36 | 13.36 | 14.86 |
| **M2** | 4 | 6 | 3.14 | 11.14 | 13.80 |
| **M3** | 6 | 7 | 2.39 | 14.39 | 20.85 |
| **M4** | 6 | 6 | 3.37 | 15.37 | 21.84 |
| **M5** | 5 | 7 | 2.19 | 12.19 | 16.48 |
| **M6** | 4 | 7 | 8.95 | 16.95 | 19.61 |
| **M7** | 5 | 6 | 2.39 | 12.39 | 16.67 |
| **M8** | 5 | 5 | 4.05 | 14.05 | 18.34 |
| **M9** | 4 | 6 | 3.38 | 11.38 | 14.05 |
| **M10** | 5 | 7 | 2.60 | 12.60 | 16.89 |
| **M11** | 4 | 7 | 8.92 | 16.92 | 19.59 |
|  |  |  |  |  |  |

The following section provides brief descriptions of each candidate model. All model text files are available in our GitHub repository.

**M1:** 3 parameters: kL1 governing enterocyte transfer of lipids, kLIP1 governing storage and recycling of lipid, and kBreakdown the clearance of chylomicrons from plasma , 4 states: Basic lipid transit model with LS → LI1 → LIP → Chp and recycling from LIP back to LI1.

**M2:** 4 parameters (kL1, kL2, kLIP1, kBreakdown). The parameters are the same as M1, but we also add the kL2 which is governing the transfer between stomach towards intestine and enterocytes. 6 states. Introduces two additional transit states (LI2 and LI3) and replaces LIP recycling with a stomach content-dependent LIP emptying mechanism. This mechanism is governed by the variable sng = (f1 + f2)^(Chp). Here f1 is input of lipids and carbohydrates and protein into the stomach, Chp is the plasma level of chylomicrons.

**M3:** 6 parameters (kL1, kL2, kLIP1, kBreakdown, kLIP2, and kd), Two new parameters; kLIP2 which governs recycling rate from the LIP state, and kd which governs the decrease of the sng state. 7 states. Same as M2, but we instead define sng as a model state.

**M4:** 6 parameters (kL1, kL2, kLIP1, kBreakdown, kLIP2, and kd), 6 states. Removes the dynamic sng state and instead calculates sng as a variable.

**M5:** 5 parameters (kL1, kLIP1, kBreakdown, kLIP2, and kd), 7 states. Same as M3 but removes the separate kL2 parameter

**M6:** 4 parameters, 6 states. (kL1, kLIP1, kBreakdown, and kd), 7 states. Same as M3 but removes the kL2 and kLIP2 parameters.

**M7:** 5 parameters, 6 states. Same as M3 but removes the T0 transit state and kl2 parameters (same as M5)

**M8:** 5 parameters, 5 states. Same as M7 but further simplifies the model by removing the LI1 state

**M9:** 4 parameters, 6 states. Same as M5 but removed the sng state (same as M4), to now be a variable.

**M10:** 5 parameters (kL1, kLIP1, kBreakdown, kLIP2, and kd), 7 states. Reintroduces the dynamic sng state, but removes the Chp-dependent component.

**M11:** 4 parameters, 7 states. Based on M10, but removes the additional kLIP2 parameter, using kLIP1 for both LIP transfer and basal emptying

**Figure S7. Agreement between candidate models and the Barrows et al. dataset.** Simulations are shown for all evaluated candidate structures, with particular emphasis on their ability to reproduce the second-meal peak.

**Table S3.** Table giving a brief description of the study intervention used for the model estimation and validation data. The description is given for each study group and each experiment.

| **Study name** | **Description of meal** | **Group** | **Experiment** | **Fat content (g)** |
| --- | --- | --- | --- | --- |
| **Pramfalk *et al.* 2015** | Overnight fasting followed by two meals. 40g Rice Krispies (Kellogg), 200g skimmed milk, and a chocolate milkshake containing 40g of olive oil. The second meal was 75g glucose. | Healthy (M) (n=11). | Two sequential mixed meals. Second meal only with glucose. | Estimated.  Breakfast: ~42.4g |
|  |  | Healthy (F) (n=11 |  |  |
| **Barrows *et al.* 2005** | Overnight fasting followed by two meals. The total energy provided by the liquid formula was 7,260±1,346 kJ, with a macronutrient profile of 54.1% of energy from carbohydrates, 32.1% from fat, and 14.1% from protein. Liquid meals. Pasteurized egg yolk, heavy whipping cream, and vegetable oil. | Healthy (M) (n=6) | Two sequential mixed meals. | Estimated ~36g.  7260kJ· 0.2390kcal ·kJ^-1^  9kcal/g |
| **Evans *et al.* 1998** | Overnight fasting followed by two meals. High-fat breakfast and lunch. The lunch was either low-fat or high fat. Breakfast: Safflower oil (55g), egg yolk, egg white, bread, jam. High-fat lunch: full-cream milk, cornflakes, full-fat ice cream, and double cream. Low-fat lunch: skimmed milk, cornflakes, and fat-free ice cream. | Healthy (M/F) (n=6) | Breakfast and  water | Given.  Breakfast: 62.9g  Water lunch: 0 g |
|  |  |  | Breakfast and low-fat lunch: | Given.  Breakfast: 62.9g  Low-fat lunch 0.9 g |
| **Heath *et al.* 2006** | Overnight fasting followed by two meals. Breakfast, consisting of 40g of Rice Krispies (Kellogg), a banana, and chocolate milkshake containing 50g of fat. The fat content of lunch was also 50g. It consisted of a cheese sandwich and a second warm chocolate milkshake. | Healthy (M) (n=8). | Breakfast and lunch | Given.  Breakfast: 51.9g  Lunch: 49.6g |
| **Milan *et al.* 2016** | Overnight fasting followed by a single meal. Mixed meal breakfast | Healthy (M/F) (n = 15)  Young ( aged 22.7±0.4 ) | Breakfast | Given.  Breakfast: 62.2g |
|  |  | Healthy (M/F) (n = 15)  Old ( aged 67.3±1.5 ) | Breakfast | Given.  Breakfast: 62.2g |
| **Vors *et al.* 2013** | Overnight fasting followed by two meals. Breakfast either as fat spread or fat emulsion. Lunch was a mixed meal. Both consisted of bread (50g), skimmed milk, and anhydrous milk fat (40g) containing 600mg tracers—either spread on bread or emulsified in skim milk. A second meal was served 5 h after breakfast. The meal contained pasta (200g), turkey (100g), butter (10g), olive oil (10g), bread (50g), and stewed fruit (100g), which provided 713 kcal (2985 kJ) with 29%, 51%, and 20% of energy as lipids (22.7g), carbohydrates (91.5g), and proteins (35.7g), respectively. | Healthy (n=9) | Two sequential mixed meals. Breakfast fat emulsion | Given.  Breakfast: 41.4g  Lunch: 22.7g |
|  |  |  | Two sequential mixed meals. Breakfast fat spread | Breakfast: 41.4g  Lunch: 22.7g |
|  |  | Obese (n=9) | Two sequential mixed meals. Breakfast fat emulsion | Breakfast: 41.4g  Lunch: 22.7g |
|  |  |  | Two sequential mixed meals. Breakfast fat spread | Breakfast: 41.4g  Lunch: 22.7g |
| **Hodson et al. 2007** | A test meal consisting of 40 g Rice Krispies (Kellogg, Manchester, U.K.), 200 g skimmed milk, and a chocolate milkshake containing 40 g of olive oil. | Healthy (F) (n=11). | Breakfast | Estimated.  Breakfast: ~42.44g |
|  |  | Insulin sensitive (M) (n=11) | Breakfast | Estimated.  Breakfast: ~42.44g |
|  |  | Insulin resistant (M) (n=11) | Breakfast | Estimated.  Breakfast: ~42.44g |

**Table S4: Model description for the M7 model.** Model parameters are presented with the best agreement to Barrows et al. 2005 dataset, with confidence interval corresponding to the best cost solution + χ2(0.95,1), and also to the agreement to all studies simultaneously with study-specific kL1 and *kBreakdown*. The model states for the M7 model are shown as zero as we always perform a steady simulation, with repeated meals, before all simulations of an intervention.

| **Barrows et al. 2005 (selection criteria)** | | | |
| --- | --- | --- | --- |
| **Parameter name** | **Value (CI)** | | **unit** |
| kL1 | 0.02177 (0.009579 - 0.03745) | | min^-1^ |
| kd | 0.06418 (0.009125 - 73.66) | | min^-1^ |
| kLIP1 | 40.51 (0.008016 - 935.7) | | min^-1^ |
| kLIP2 | 7.768 (0.002093 - 90.19) | | min^-1^ |
| kBreakdown | 0.1113 (0.09197 - 0.1426) | | min^-1^ |
| **All studies combined (Fig. 2)** | | | |
| **Parameter name (common)** | **Value (CI) (common)** | | **unit** |
| kd | 42.67 (25.3 - 76.53) | | min^-1^ |
| kLIP1 | 0.2223 (0.1529 - 0.3774) | | min^-1^ |
| kLIP2 | 0.07231 (0.06067 - 0.1034) | | min^-1^ |
| **Study Name** | **kBreakdown (Value (CI)** | **kL1 (Value (CI)** |  |
| Parmfalk_2015 | 0.04472 (0.02976 - 0.05015) | 0.01544 (0.009959 - 0.02039) | min^-1^ |
| Barrows_2005 | 0.1111 (0.1021 - 0.1241) | 0.01963 (0.01752 - 0.02361) | min^-1^ |
| Parmfalk_2015_f | 0.07958 (0.06395 - 0.1032) | 0.01822 (0.01648 - 0.02112) | min^-1^ |
| Milan_2016_y | 0.1327 (0.1238 - 0.1546) | 0.02771 (0.02277 - 0.03601) | min^-1^ |
| Milan_2016_o | 0.1054 (0.09857 - 0.1205) | 0.01971 (0.01743 - 0.02334) | min^-1^ |
| Heath_2006 | 0.09457 (0.0847 - 0.1082) | 0.02077 (0.01691 - 0.02731) | min^-1^ |
| Evans_1998_lf | 0.0963 (0.08834 - 0.1018) | 0.01338 (0.01181 - 0.01704) | min^-1^ |
| Evans_1998_wa | 0.09774 (0.08657 - 0.1095) | 0.02779 (0.02279 - 0.0349) | min^-1^ |
| Vors_2013_sp | 0.08935 (0.08199 - 0.09874) | 0.02355 (0.02051 - 0.02822) | min^-1^ |
| Vors_2013_em | 0.08678 (0.07084 - 0.1143) | 0.01606 (0.01327 - 0.01993) | min^-1^ |
| Vors_2013_ob_sp | 0.03256 (0.02495 - 0.03706) | 0.008583(0.007243 - 0.009617) | min^-1^ |
| Vors_2013_ob_em | 0.08088 (0.06778 - 0.09666) | 0.02227 (0.018 - 0.02486) | min^-1^ |
| **State name** | **initial condition** | |  |
| sng | 0 | | a.u. |
| LS | 0 | | mmol |
| T | 0 | | mmol |
| LI1 | 0 | | mmol |
| LIP | 0 | | mmol |
| Chp | 0 | | mmol |

**Figure S8: M7 model prediction of all studies when only trained to the Barrows et al. 2005 dataset.** *The model can predict* *some datasets qualitatively well but fails to predict the magnitude of the chylomicron response in several cases.* **(A)** *Pramfalk et al. 2015 male cohort validation cost = 8.74 < Tχ2 = 21.026.* **(B)** *Pramfalk et al. 2015 female cohort validation cost = 2.310 < Tχ2 = 21.026.* **(C)** *Milan et al. 2016 young cohort validation cost = 9.474 < Tχ2 = 12.592.* **(D)** *Milan et al. 2016 old cohort validation cost = 15.801 > Tχ2 = 12.592.* **(E)** *Heath et al. 2006 healthy cohort validation cost = 62.748 > Tχ2 = 18.307.* **(F)** *Evans et al. 1998 high-fat cohort validation cost = 150.433 > Tχ2 = 18.307.* **(G)** *Evans et al. 1998 low-fat cohort validation cost = 34.287 > Tχ2 = 18.307.* **(H)** *Evans et al. 1998 water cohort validation cost = 60.788 > Tχ2 = 18.307.* ***(I)*** *Vors et al. 2013 normal spread* *cohort validation cost = 47.780 > Tχ2 = 16.919.* **(J)** *Vors et al. 2013 normal emulsion* *cohort validation cost = 31.990 > Tχ2 = 16.919.* **(K)** *Vors et al. 2013 obese spread. cohort validation cost = 51.297 > Tχ2 = 16.919.* **(L)** *Vors et al. 2013 obese emulsion cohort validation cost = 33.399 > Tχ2 = 16.919.* **(M)** *Hodson et al. 2007 female cohort validation cost = 7.889 < Tχ2 = 12.592.* **(N)** *Hodson et al. 2007 insulin sensitive men cohort validation cost = 28.506 > Tχ2 = 12.592.* **(O)** *Hodson et al. 2007 insulin resistant men cohort validation cost = 41.052 > Tχ2 = 12.592.*

 **Figure S9: Profile likelihood analysis for the M7 model when finding agreement to the Barrows et al. 2005 data.** For this analysis we used the global ESS (MEIGO toolbox) search for each parameter step. The blue line indicates the best cost-value, and the red line is the best cost solution + χ2(0.95,1). Note the different y-axis scales between kd, kLIP1, and KLIP2 compared to kL1 and kBreakdown.

**Figure S10: Profile likelihood analysis for the M7 model when finding agreement to the Barrows et al. 2005 data near the best-fit solution.** For this analysis we used the local *fmincon* (MATLAB internal) search for each parameter step.
